# Supplementary material for: The Impact of Lower-Strength Alcohol Products on Alcohol Purchases by Spanish Households
Source: Nutrients. 2022 Aug 19;14(16):3412. doi: 10.3390/nu14163412 (PMC9413452; doi:10.3390/nu14163412)
Supplement: Supplementary file 1 [file nutrients-14-03412-s001.zip › nutrients-1848638-supplementary.pdf]

Supplement Table S1 Divisions of households by socio-demographic characteristics. (N=18,954 households).

Group	Categories	Number of households within each group	Distribution within each group (%)
Age of the main shopper (years)	18-34	3668	19.4
	35-49	7690	40.6
	50-64	5634	29.7
	65+	1962	10.4
Social grade[a]	Medium-High and High	3397	17.9
	Medium-Medium	6375	33.6
	Medium-Low	5081	26.8
	Low	4101	21.6
Autonomous Community	Andalusia	3319	17.5
	Aragon	547	2.9
	Asturias	444	2.3
	Balearic Islands	416	2.2
	Basque Country	788	4.2
	Canary Islands	838	4.4
	Cantabria	234	1.2
	Castile and León	1277	6.7
	Castilla- La Mancha	824	4.3
	Catalonia	3036	16.0
	Community of Madrid	2783	14.7
	Extremadura	467	2.5
	Galicia	1083	5.7
	La Rioja	136	.7
	Murcia	552	2.9
	Navarre	214	1.1
	Valencian Community	1996	10.5

[a]Based on 20 variables, using Multiple Correspondences Analysis (MCA), households were factored into one of four groups as follows:

Low Grade
Head of family:
Owners of agriculture, livestock and fisheries
Employees in agriculture, livestock and fisheries
No studies
Other: students, retired, etc.
Equipment:
No internet
No computer
No mobile phone
No car
No microwave
No vacuum cleaner
No dishwasher
No stereo
1 TV

Medium-Low Grade
Head of family:
Lower levels of employment
Primary school
Equipment:
No dishwasher
1 Bathroom
1 car
1 TV set
No pay TV
No electric toothbrush
No housekeeper
No second residence

Medium-Medium Grade
Head of family:
Mid management
Office employees
Industry and commerce employees
Secondary school
Equipment:
Robotic Vacuum cleaner
Dishwasher
Stereo
2+ cars
2+ TV sets
2+ bathrooms
Electric toothbrush

Medium-High and High Grade
Head of family:
High management
Mid management
Self-employed
University
Equipment:
Housekeeper
Secondary residence
3+ TV sets
2+ cars

Interrupted Time Series Analyses

Hypothesis 1: The purchases of no-alcohol beers substitute the purchase of all other higher-strength beers, leading to less grams of alcohol purchased.

Sample: Households that had made at least one purchase of no-alcohol beer and at least one purchase of any other beer, with the first purchase of the no-alcohol beer occurring more than 90 days after the first purchase of any other beer (n=4115 households).

The dependent variables are:
1. Grams of all purchased alcohol, per adult per household per day of purchase (primary outcome).
2. Volume of purchases of all beers with an ABV >0.5%, per adult per household per day of purchase (secondary outcome).

The independent variables are:
1. The event, the date of purchase of beer with an ABV ≤0.5%, entered as a dummy variable coded with 0 for each day before the event and with 1 for each day from the event forwards. All households were adjusted to the same event day by, for each household, setting the day of first purchase of the no-alcohol beer as day 1, numbering all other days as before (minus days) or after (plus days)
2. The Alcohol by Volume (ABV%) of all purchased beer with an ABV >0.5%, averaged per study day.

Distribution of primary outcome
The primary outcome, grams of all purchased alcohol, per adult per household per day of purchase across all study days was normally distributed, Supplement Figure S1.

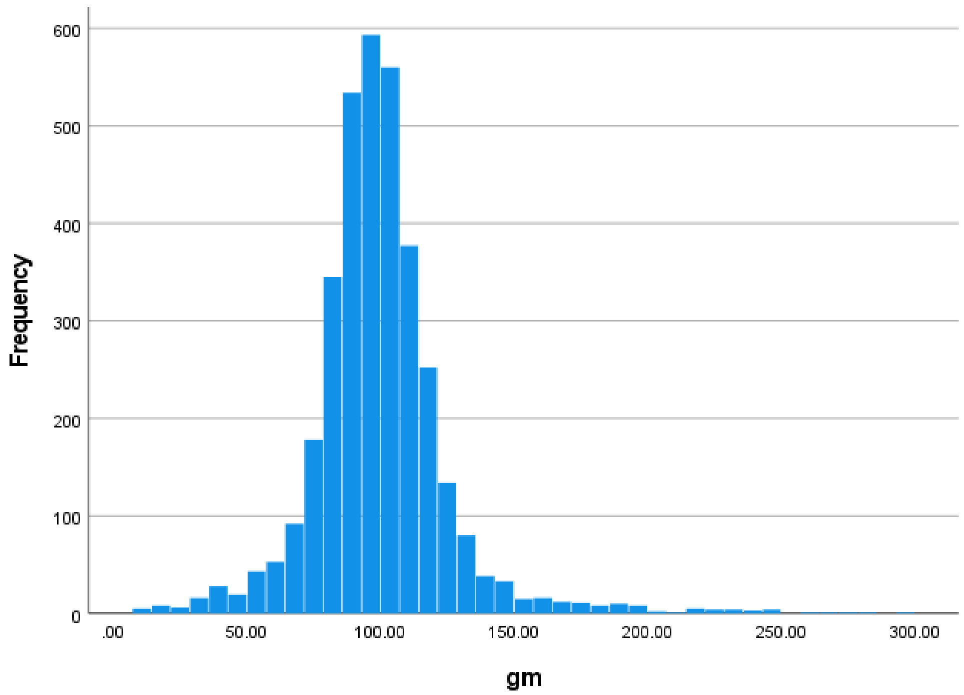

Supplement Figure S1 Frequency distribution of grams of all purchased alcohol per adult per household per day of purchase across all study days (gm).

Seasonal adjustment
Given the weekly variation in purchases (see Supplement Figure S7), we adjusted the data by week using the Census Method I, ratio-to-moving-average method (Makridakis S, Wheelwright SC, McGee VE. *Forecasting: methods and applications*. New York: John Wiley and Sons, 1983; McLaughlin RL. *Forecasting techniques for decision making*. Rockville, MD: Control Data Management Institute, 1984).

Time series
We used a time series modeler function to estimate best fitting non-seasonal and seasonal ARIMA models that: a) specify degrees of differencing and/or a square root or natural log transformation to ensure a stationary series; and, b) specify autoregressive and moving average orders. This eliminated the need to identify an appropriate ARIMA model through trial and error: (ftp://public.dhe.ibm.com/software/analytics/spss/documentation/statistics/26.0/en/client/Manuals/IBM_SPSS_Forecasting.pdf.) As the ARIMA model (0,0,0,) (0,0,0) was stationary with no autocorrelation (Supplement Figure S2), we used a generalized linear model for our estimates, with the following SPSS syntax:

```
GENLIN grams BY event WITH abv
 /MODEL event abv INTERCEPT=YES
 DISTRIBUTION=NORMAL LINK=IDENTITY
 /CRITERIA SCALE=MLE COVB=MODEL PCONVERGE=1E-006(ABSOLUTE) SINGULAR=1E-012
ANALYSISTYPE=3(WALD)
   CILEVEL=95 CITYPE=WALD LIKELIHOOD=FULL
 /MISSING CLASSMISSING=EXCLUDE
 /PRINT CPS DESCRIPTIVES MODELINFO FIT SUMMARY SOLUTION
```

Where:

- grams= grams of all purchased alcohol per adult per household per day of purchase per study day
- event = the date of purchase of beer with an ABV ≤0.5%, entered as a dummy variable coded with 0 for each day before the event and with 1 for each day from the event forwards
- ABV=the mean ABV of all purchased beer with an ABV>0.5% per study day.

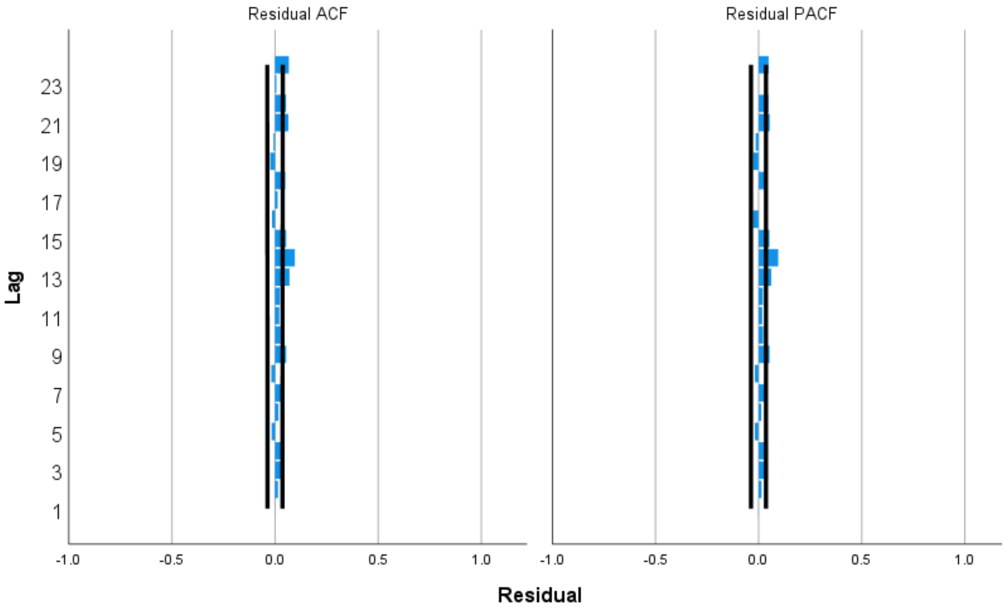

Supplement Figure S2. Residual ACF and PACF of time series for grams of all purchased alcohol per adult per household per day of purchase.

Impact of household characteristics

To study the impact of household characteristics (see Supplement Table S1) for each of the three main groups (age of main shopper, social grade of household, and Autonomous Community in which the household is located), we ran three separate models for each household group, adding the household characteristic as a factor, with an interaction term, event*household characteristic as follows:

```
GENLIN grams BY characteristic event WITH abv
  /MODEL event characteristic event*characteristic abv INTERCEPT=YES
 DISTRIBUTION=NORMAL LINK=IDENTITY
  /CRITERIA SCALE=MLE COVB=MODEL PCONVERGE=1E-006(ABSOLUTE) SINGULAR=1E-012
ANALYSISTYPE=3(WALD)
    CILEVEL=95 CITYPE=WALD LIKELIHOOD=FULL
  /MISSING CLASSMISSING=EXCLUDE
  /PRINT CPS DESCRIPTIVES MODELINFO FIT SUMMARY SOLUTION
```

Hypothesis 2: The purchases of no-alcohol wines substitute the purchase of all other higher-strength wines, leading to less grams of alcohol purchased.

Sample: Households that had made at least one purchase of no-alcohol wine and at least one purchase of any other wine, with the first purchase of the no-alcohol wine occurring more than 90 days after the first purchase of any other wine. N=1271 households.

The dependent variables are:

3. Grams of all purchased alcohol, per adult per household per day of purchase (primary outcome).
4. Volume of purchases of all wines with an ABV >0.5%, per adult per household per day of purchase (secondary outcome).

The independent variables are:

3. The event, the date of purchase of wine with an ABV ≤0.5%, entered as a dummy variable coded with 0 for each day before the event and with 1 for each day from the event forwards. All households were adjusted to the same event day by, for each household, setting the day of first purchase of the no-alcohol wine as day 1, numbering all other days as before (minus days) or after (plus days)
4. The Alcohol by Volume (ABV%) of all purchased wine with an ABV >0.5%, averaged per study day.

Distribution of primary outcome

The primary outcome, grams of all purchased alcohol, per adult per household per day of purchase across all study days was normally distributed, Supplement Figure S3.

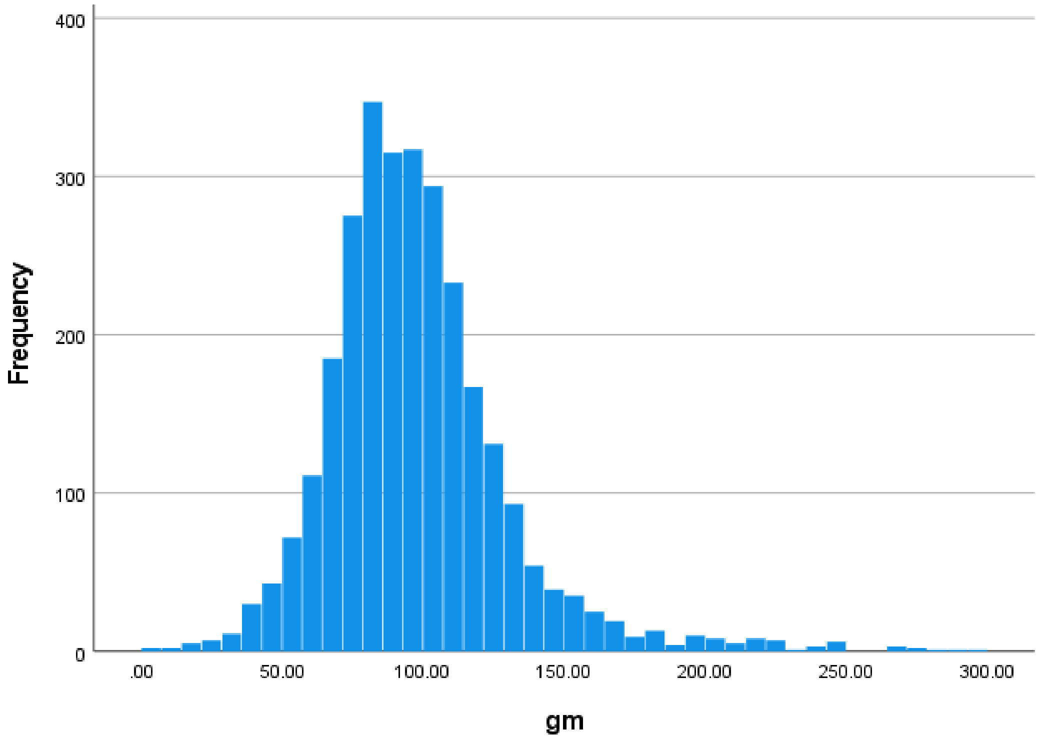

Supplement Figure S3 Frequency distribution of grams of all purchased alcohol per adult per household per day of purchase across all study days (gm).

Seasonal adjustment
Given the weekly variation in purchases (see Supplement Figure S8), we adjusted the data by week using the Census Method I, ratio-to-moving-average method (Makridakis S, Wheelwright SC, McGee VE. *Forecasting: methods and applications*. New York: John Wiley and Sons, 1983; McLaughlin RL. *Forecasting techniques for decision making*. Rockville, MD: Control Data Management Institute, 1984).

Time series
We used a time series modeler function to estimate best fitting non-seasonal and seasonal ARIMA models that: a) specify degrees of differencing and/or a square root or natural log transformation to ensure a stationary series; and, b) specify autoregressive and moving average orders. This eliminated the need to identify an appropriate ARIMA model through trial and error: (ftp://public.dhe.ibm.com/software/analytics/spss/documentation/statistics/26.0/en/client/Manuals/IBM_SPSS_Forecasting.pdf.) As the ARIMA model (0,0,0,) (0,0,0) was stationary with no autocorrelation (Supplement Figure S4), we used a generalized linear model for our estimates, with the following SPSS syntax:

```
GENLIN grams BY event WITH abv
  /MODEL event abv INTERCEPT=YES
 DISTRIBUTION=NORMAL LINK=IDENTITY
  /CRITERIA SCALE=MLE COVB=MODEL PCONVERGE=1E-006(ABSOLUTE) SINGULAR=1E-012
ANALYSISTYPE=3(WALD)
    CILEVEL=95 CITYPE=WALD LIKELIHOOD=FULL
  /MISSING CLASSMISSING=EXCLUDE
  /PRINT CPS DESCRIPTIVES MODELINFO FIT SUMMARY SOLUTION
```

Where:
- grams= grams of all purchased alcohol per adult per household per day of purchase per study day
- event = the date of purchase of wine with an ABV ≤0.5%, entered as a dummy variable coded with 0 for each day before the event and with 1 for each day from the event forwards
- ABV=the mean ABV of all purchased wine with an ABV>0.5% per study day.

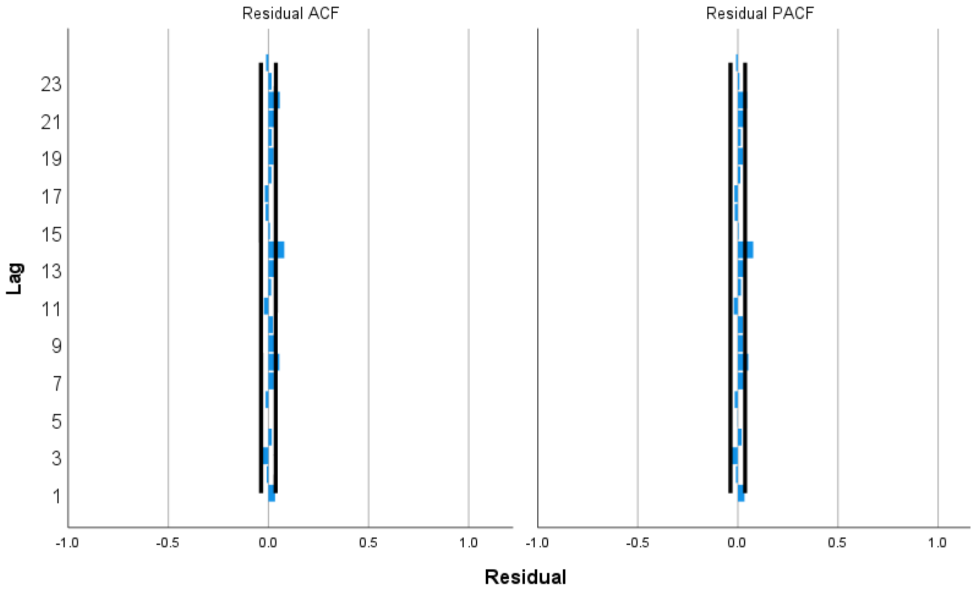

Supplement Figure S4. Residual ACF and PACF of time series for grams of all purchased alcohol per adult per household per day of purchase.

Impact of household characteristics

To study the impact of household characteristics (see Supplement Table S1) for each of the three main groups (age of main shopper, social grade of household, and Autonomous Community in which the household is located), we ran three separate models for each household group, adding the household characteristic as a factor, with an interaction term, event*household characteristic as follows:

```
GENLIN grams BY characteristic event WITH abv
  /MODEL event characteristic event*characteristic abv INTERCEPT=YES
 DISTRIBUTION=NORMAL LINK=IDENTITY
  /CRITERIA SCALE=MLE COVB=MODEL PCONVERGE=1E-006(ABSOLUTE) SINGULAR=1E-012
ANALYSISTYPE=3(WALD)
   CILEVEL=95 CITYPE=WALD LIKELIHOOD=FULL
 /MISSING CLASSMISSING=EXCLUDE
 /PRINT CPS DESCRIPTIVES MODELINFO FIT SUMMARY SOLUTION
```

Hypothesis 3: The purchases of 20% ABV variants of same-branded whisky and gin substitute the purchase of regular-strength variants, leading to less grams of alcohol purchased.

Sample: Households that had made at least one purchase of any variant of the whisky or gin (n=1208 households), split into those that had purchased a 20% ABV variant (n=30) and those that had not (n=1178).

The dependent variables are:
1. The total grams of alcohol purchased within all spirits (primary outcome).
2. The volume of purchases of the regular-strength variants (secondary outcome).
3. The total grams of alcohol purchased within any of the variants (secondary outcome).
4. The total grams of alcohol purchased within any spirits other than the branded variants of the whisky or gin (to examine potential shifts from other spirit products to the 20% variants) (secondary outcome).
5. The ABV of all spirits purchased (secondary outcome).
6. Grams of all purchased alcohol, per adult per household per day of purchase (secondary outcome).

The independent variables are:
1. The event, the date of introduction of the 20% ABV variants as evidenced by the first household purchase of either product. The event was entered as a dummy variable coded with 0 for each day before the event and with 1 for each day from the event forwards.
2. "Whether or not" the household (within all 1208 households that had made at least one purchase of any of the variants of the whisky or gin) had made at least one purchase of a 20% ABV variant, dummy coded as 0 (had not) or 1 (had).
3. Interaction term event*"whether or not".

Distribution of primary outcome
The primary outcome, grams of all purchased alcohol within spirits, per adult per household per day of purchase across all study days was normally distributed, Supplement Figure S5.

Seasonal adjustment
Given the annual variation in purchases (see Supplement Figure S9), we seasonally adjusted the data by year using the Census Method I, ratio-to-moving-average method (Makridakis S, Wheelwright SC, McGee VE. *Forecasting: methods and applications*. New York: John Wiley and Sons, 1983; McLaughlin RL. *Forecasting techniques for decision making*. Rockville, MD: Control Data Management Institute, 1984).

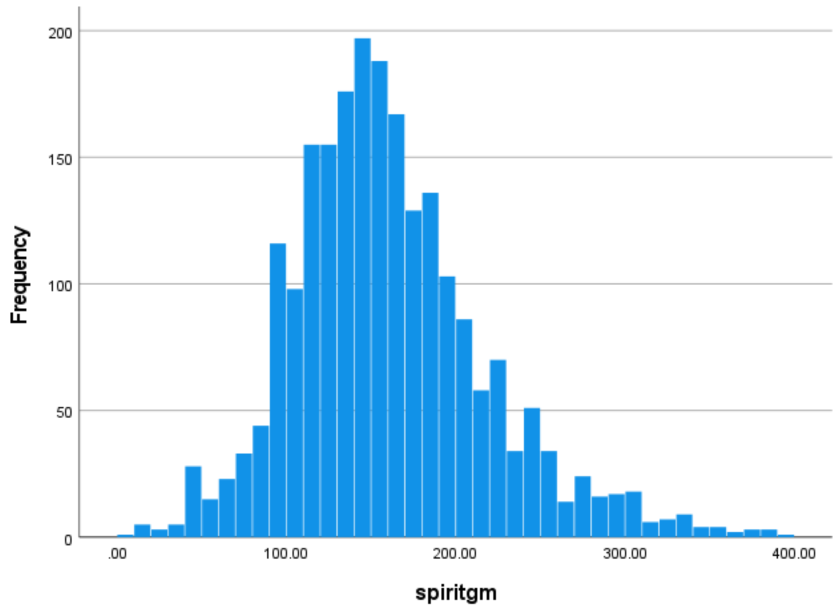

Supplement Figure S5 Frequency distribution of grams of all purchased alcohol within spirits per adult per household per day of purchase across all study days (gm).

Time series
We used a time series modeler function to estimate best fitting non-seasonal and seasonal ARIMA models that: a) specify degrees of differencing and/or a square root or natural log transformation to ensure a stationary series; and, b) specify autoregressive and moving average orders. This eliminated the need to identify an appropriate ARIMA model through trial and error: (ftp://public.dhe.ibm.com/software/analytics/spss/documentation/statistics/26.0/en/client/Manuals/IBM_SPSS_Forecasting.pdf.) As the ARIMA model (0,0,0,) (0,0,0) was stationary with no autocorrelation (Supplement Figure S6), we used a generalized linear model for our estimates. However, given the small number of households and purchases, in particular for those that had made at least one purchase of a 20% ABV variant of Ballantine's Scotch whisky and Beefeater gin, we used the time series modeler function, that, based on the available data, predicted values across the missing study days. We used these predicted values in the regression models, with the following SPSS syntax:

```
GENLIN pre_spiritgrams BY householdgroup event
 /MODEL householdgroup event householdgroup*event INTERCEPT=YES
 DISTRIBUTION=NORMAL LINK=IDENTITY
 /CRITERIA SCALE=MLE COVB=MODEL PCONVERGE=1E-006(ABSOLUTE) SINGULAR=1E-012
ANALYSISTYPE=3(WALD)
   CILEVEL=95 CITYPE=WALD LIKELIHOOD=FULL
 /MISSING CLASSMISSING=EXCLUDE
 /PRINT CPS DESCRIPTIVES MODELINFO FIT SUMMARY SOLUTION
```

Where:

- Pre_spiritgrams= predicted value of grams of all purchased alcohol within spirits per adult per household per day of purchase per study day
- householdgroup dummy coded as 0 (had not purchased any 20% ABV variant) or 1 (had purchased)
- event = the date of introduction of the 20% ABV variants dummy coded with 0 for each day before the event and with 1 for each day from the event forwards

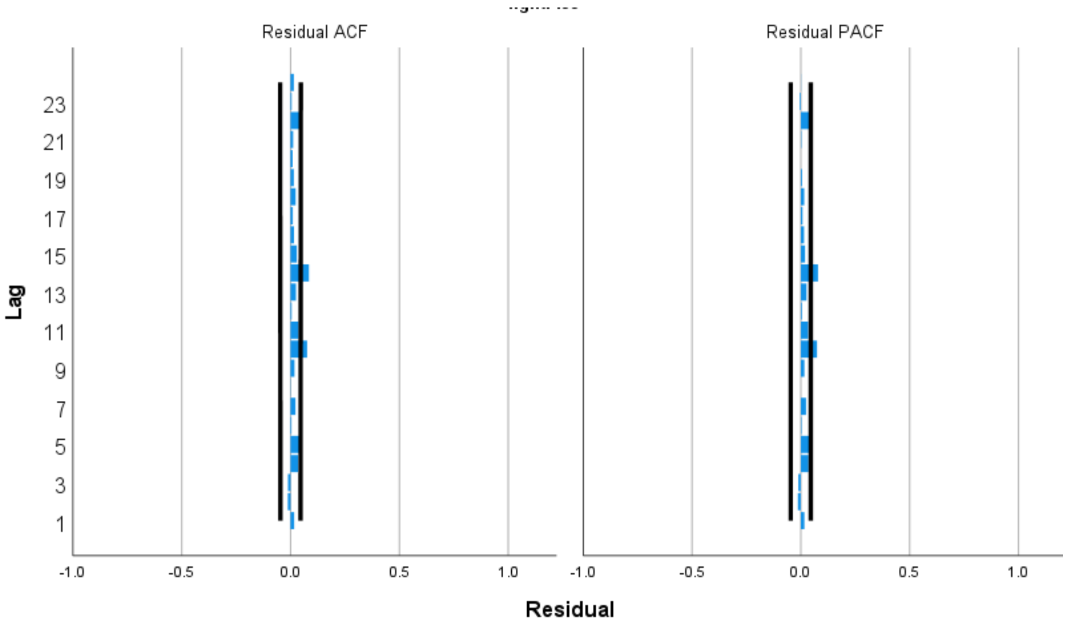

Supplement Figure S6. Residual ACF and PACF of time series for grams of all purchased alcohol within spirits per adult per household per day of purchase.

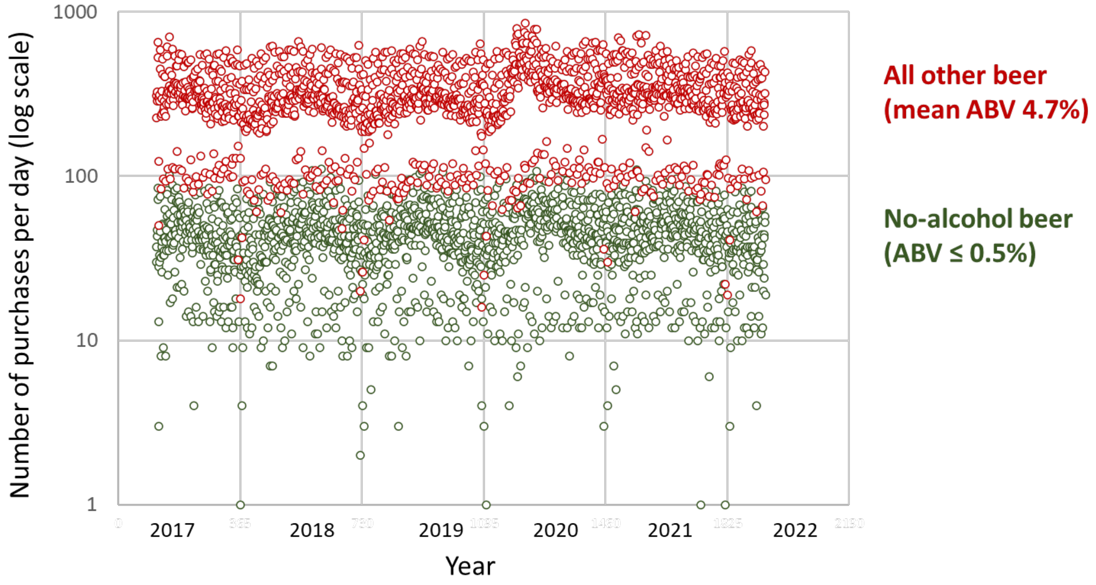

Supplement Figure S7 Plots of number of separate purchases (log[10] scale) of beers for each calendar day by ABV group summed across 18,954 Spanish households. Data points: daily. For each ABV group, lower values are for days with less purchases (Sundays). The increase in number of household purchases during 2020 was associated with COVID-19 lockdowns. A regression model with number of purchases of no-alcohol beer as dependent variable and time (days) as independent variable found a stable trend (coefficient=0.00083, 95%CI=-0.00095 to 0.0026).

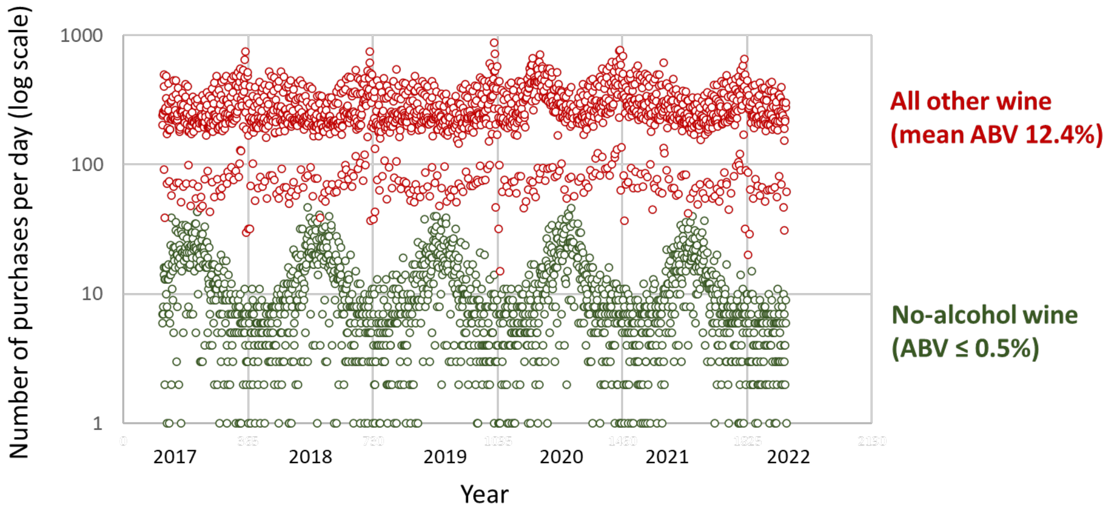

Supplement Figure S8 Plots of number of separate purchases (log[10] scale) of wines for each calendar day by ABV group summed across 18,954 Spanish households. Data points: daily. For each ABV group, lower values are for days with less purchases (Sundays). A regression model with number of purchases of no-alcohol wine as dependent variable and time (days) as independent variable found a very slight decrease over time (coefficient=-0.00229, 95%CI=-0.00301 to -0.00158).

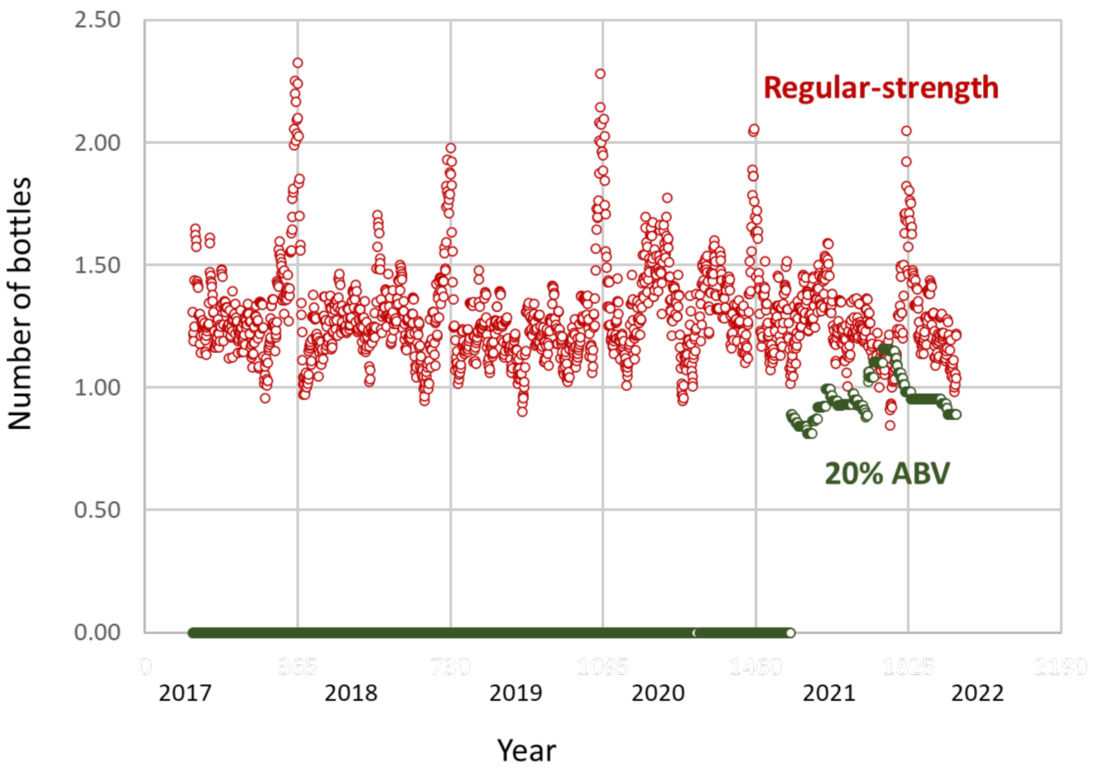

Supplement Figure S9 Plots of mean number of purchased bottles per day of purchase (adjusted to 700ml) per study day (adjusted over each week) of any variant of the whisky or gin for regular strength products and 20% ABV products summed across 18,954 Spanish households). Data points: daily, smoothed.
